# Diffusion enables integration of heterogeneous data and user-driven learning in a desktop knowledge-base

**DOI:** 10.1371/journal.pcbi.1009283

**Published:** 2021-08-11

**Authors:** Tomasz Konopka, Sandra Ng, Damian Smedley

**Affiliations:** William Harvey Research Institute, Queen Mary University of London, London, United Kingdom; University of Colorado School of Medicine, UNITED STATES

## Abstract

Integrating reference datasets (e.g. from high-throughput experiments) with unstructured and manually-assembled information (e.g. notes or comments from individual researchers) has the potential to tailor bioinformatic analyses to specific needs and to lead to new insights. However, developing bespoke analysis pipelines from scratch is time-consuming, and general tools for exploring such heterogeneous data are not available. We argue that by treating all data as text, a knowledge-base can accommodate a range of bioinformatic data types and applications. We show that a database coupled to nearest-neighbor algorithms can address common tasks such as gene-set analysis as well as specific tasks such as ontology translation. We further show that a mathematical transformation motivated by diffusion can be effective for exploration across heterogeneous datasets. Diffusion enables the knowledge-base to begin with a sparse query, impute more features, and find matches that would otherwise remain hidden. This can be used, for example, to map multi-modal queries consisting of gene symbols and phenotypes to descriptions of diseases. Diffusion also enables user-driven learning: when the knowledge-base cannot provide satisfactory search results in the first instance, users can improve the results in real-time by adding domain-specific knowledge. User-driven learning has implications for data management, integration, and curation.

## Author summary

Biological datasets can be too large to unravel without computational tools and, at the same time, too small to benefit from machine-learning approaches that require large data volumes to train. Analyses of niche datasets are also hindered by the difficulty of incorporating knowledge from domain experts into practical algorithms. In this work, we argue that such challenges may be addressed by data integration platforms that can learn in real-time from users. We support the argument with an implementation of a practical, general-purpose, tool. We show that its search capabilities can solve common bioinformatic tasks such as gene-set analysis and matching genes and phenotypes to diseases, which are usually tackled using statistical methods and bespoke algorithms. Importantly, our tool leaves domain experts full control to modulate search results in transparent, biologically meaningful ways. It also allows users to improve search outputs if the default results are incomplete. These features extend the capabilities of existing knowledge-bases and empower domain experts to tailor data exploration to their needs.

## Introduction

Biological assays measure diverse entities that range from single-molecules to entire ecosystems. This heterogeneity provides opportunities to test hypotheses and to formulate new ones, but such data can be difficult to navigate and to integrate in practice. Web portals demonstrate the power of interactive access to complex data [1–3]. However, portals are still unavailable for most research domains, especially in emerging subjects. Some of the challenges include assembling relevant data in a coherent format and implementing interfaces that are at once powerful, responsive, and user-friendly [4]. Further complications arise when adapting an existing system to incorporate additional data or to fine-tune performance.

Given that maintaining bespoke data portals for individual research projects is impractical, the alternative may be to utilize general-purpose components instead. Any generalised framework built to support multiple applications will trade decreased performance for increased versatility. A pertinent issue becomes how this balance should be set in the context of biological data, but two observations point in favor of increased versatility. First, biological datasets often have limited sizes and lack ground-truth annotations. They are not always suitable for processing with pre-trained machine-learning models and are not sufficient to train new models de-novo. Facilitating data exploration should thus prioritize transparent approaches that can provide insight with little preparation. Second, in tasks where multiple methods are available—for example gene-module detection—systematic evaluations suggest that one method does not outperform in all situations [5]. This suggests that specialized models, while useful in their area of applicability, are not sufficient for exploratory analyses; it is beneficial to explore a range of alternatives. In addition to these data- and application-centric arguments, we also note that explorative analyses of biological data are performed by expert users with extensive knowledge. A general-purpose framework may therefore be advantageous if it could incorporate domain-specific information by learning in real-time from its users.

In this work, we explore the feasibility of a general-purpose knowledge-base for biological data. To focus the discussion, we set aside rich-media such as images, audio, and video, and observe that much data in molecular and medical biology can be represented as structured and quasi-structured text. Examples include abstracts from journal articles, concept definitions encoded in dictionaries and ontologies, gene sets based on curated resources or from measurement pipelines, and even sequences of nucleic and amino acids. We explore treating such collections of text from a single computational perspective, even if they are distinct in biological meaning. We investigate to what extent a general approach can reproduce results from specialized analyses, facilitate integration of diverse datasets, and adapt in response to feedback from users.

A key component of text mining is the encoding of text into numeric representations in order to enable calculations. Encodings based on k-mers, which are simple to implement and to understand, can capture relatedness between words while tolerating variations in spelling [6]. K-mers have been used to index biological datasets such as databases of DNA and protein sequences [7], and such indexing and retrieval strategies can be extended to arbitrary text [8]. More sophisticated encodings have been used to perform computations like composition [9] and neural-networks have been trained for text classification [10,11], including using biomedical corpora as training sets [12,13]. However, neural networks require considerable computational effort to train and to update, and, even when they perform well in benchmark analyses, they achieve performance only a few percent higher than simpler k-mer or bag-of-words approaches. Thus, the simpler approaches based on k-mers offer a solid foundation on which to construct a framework for general-purpose data exploration.

A challenge associated with treating text as a collection of k-mers is that data tends to be represented as sparse numeric vectors. Sparse data is problematic because it can be difficult to quantify similarities between vectors when they have few features in common. This issue appears in applications in genomics such as analyses of somatic mutations in cancer—where sparsity is a consequence of the relative rarity of somatic events in cancer genomes—and in single-cell sequencing datasets—where sparsity is a result of the limited number of DNA or RNA molecules in individual cells. Imputation has proved an effective technique because it provides additional features that facilitate comparisons between otherwise sparse vectors [14,15]. A specific approach to imputation is data diffusion, which is inspired by physical diffusion that transfers molecules from regions of high concentration to nearby regions of low concentration. Data diffusion instead transfers weight from k-mers observed in real data to other k-mers that were seen to co-occur in some reference dataset. In addition to being transparent and biologically interpretable, diffusion processes can also be tuned in a computationally efficient manner. Thus, diffusion can at once help overcome difficulties associated with sparse data and enable a knowledge-base to absorb feedback from users.

In this work, we demonstrate a practical approach that integrates text-based datasets, provides querying mechanisms suited to bioinformatic questions, and personalizes results based on user feedback. We show its utility in two practical bioinformatic applications—gene-set analysis and ontology mapping. In the process we produce competitive solutions to well-established bioinformatic tasks. We then demonstrate possibilities for exploring integrated data in an interactive manner, including through multi-modal queries.

## Results

### Nearest-neighbor algorithms augment data stores

**Fig 1:**
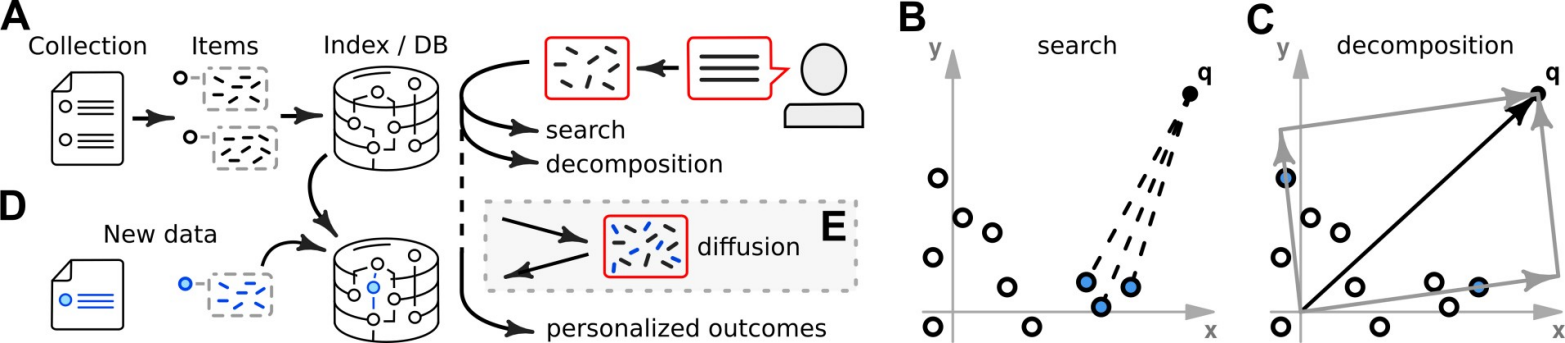
Knowledge-base setup and user interactions. **(A)** A collection of data is parsed into data items, which are split into k-mers. Data items are then inserted into a database and indexed. User queries are also parsed into k-mers and then processed using search and decomposition algorithms. **(B)** Schematic of a search query in a feature space. A user query, q, is compared to a collection of items (circles). The search algorithm reports a number of items that are closest to the query (blue circles). **(C)** Schematic of query decomposition, which returns distinct data items that, together, can reproduce the query. **(D)** New data items are inserted into the database in a way that preserves their status as user-specific. **(E)** A diffusion process augments user queries with imputed features, including features based on user-specific data, and provides a mechanism to personalize outcomes.

With the aim to analyze many types of biomedical data in a range of applications, we set up software that combines a heterogenous data store with algorithms based on nearest-neighbors (Methods). Our approach starts by parsing a collection of text data into individual records and subsequently into k-mers (Fig 1A). K-merization is a step that splits long words into their constituent parts, e.g. parsing the word ‘vascular’ into 6-mers ‘vascul’, ‘ascula’, and ‘scular’. We store data items in their original form (as text) in a database and also as sparse vectors of k-mers in a separate nearest-neighbor (NN) index [16]. Two primary mechanisms for querying the data are provided: search and decomposition. Both accept text as input, utilize the NN index, and return records from the data store. Search is a straightforward lookup of data items in the NN index. It ranks items that are most similar to the user query and thus often returns several hits that are similar to each other (Fig 1B). Decomposition, in contrast, is a custom iterative algorithm (Methods). It reports a combination of records from the database that, together, cover the features present in the input (Fig 1C).

A key feature of our approach is its ability to tune the behavior of the knowledge-base at run-time and in real-time. This is motivated by real-world situations where a user might observe that the default results are not well-adapted to a specific task. In such cases, our system can accept new data items from the user and incorporate them into the data store (Fig 1D). The internal architecture separates original and added data items in a strict manner. This helps preserve integrity of original data and also allows the data store to perform updates almost instantaneously. Adding new data into a database is not remarkable in itself, but becomes a key ingredient to our approach when coupled with data imputation (Methods, Fig 1E). We implement an algorithm that we call data diffusion due to its similarity to physical diffusion, i.e. the motion of particles from regions of high concentration to regions of low concentration. The algorithm considers k-mers in a user query and shifts some of their weight toward other k-mers that co-occured in reference datasets. Diffusion can be set to employ only original data, only user-specific data, or a weighted combination. Passing the augmented query to search and decomposition can result in different hits. Thus, because diffusion can incorporate information from recently added data, users can immediately observe changes in search and decomposition outcomes as a result of training.

Importantly, the data store and our algorithms only process text. This design choice foregoes optimizations that might be possible were the software restricted to, for example, genes or other entities from fixed vocabularies. However, text offers users the flexibility to prepare data in a text editor for batch processing or in a text box in a graphical user interface. K-merization makes the algorithms naturally resistant to spelling errors. Moreover, the algorithms are not tied to any specific data type and do not utilize sophisticated natural-language processing. They can thus be used in a range of applications. For illustration, we performed calculations using gene sets, ontologies, and disease annotations.

### Decomposition of gene sets extracts distinct concepts

**Fig 2:**
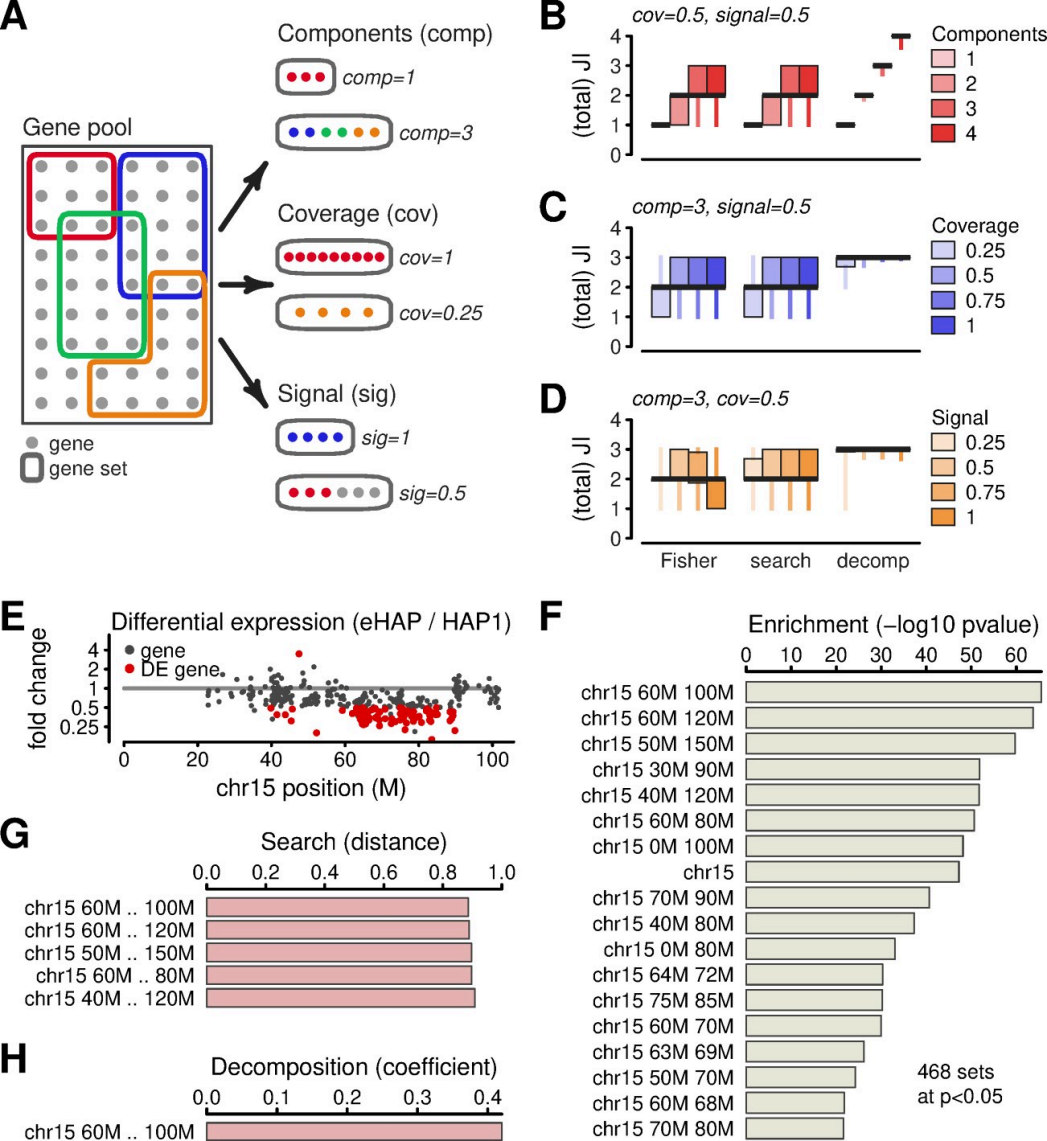
Applications of search and decomposition algorithms on gene sets. **(A)** Schematic of synthetic gene sets for benchmarking calculations. The gene pool contains all human genes and sets are based on GO annotations. Benchmarking datasets are generated by randomly selecting genes from one or more sets. Generation strategies include components (taking genes from one or more GO set), coverage (taking all genes from a set, or fractions thereof), signal (augmenting genes from GO sets with randomly selected genes). **(B-D)** Results of benchmarking calculations performed using three computational methods—the Fisher statistical test, and search and decomposition powered by nearest-neighbor searches. Vertical axes show sums of Jaccard Indexes between reported and expected gene sets. This score counts the number of correctly identified components. **(E)** Visualization of differentially-expressed genes in the eHAP cell line along one of the chromosomes. **(F)** Output of hypeR gene set enrichment analysis, comparing DE genes with a collection of sets that contain genes from genomic regions of various sizes. **(G)** Output from gene set search. **(H)** Output from gene set decomposition.

One of the bioinformatic applications that relies on search is gene-set analysis. Given genes observed in an experiment, gene-set analysis quantifies similarities of the hits against a collection of other sets. Many methods have been developed for this task, including some specific to transcriptomics [17,18]. To understand to what extent a general-purpose tool can be used for gene set analysis, we set up benchmarking calculations based on the Gene Ontology (GO) [19]. We created synthetic gene sets through three distinct strategies (Fig 2A). One strategy combined genes from multiple GO sets into a single benchmark (number of components from one to four). This simulates data from systems characterized by more than one aberration or activated process. Another strategy modulated the proportion of genes that were transferred from the GO annotations into synthetic benchmarks (coverage from 25% to 100%). A third strategy supplemented GO-based gene sets with randomly-picked genes (signal from 25% to 100%). By considering 1000 examples for each combination of these effects, we created 64,000 synthetic sets. We then matched them against the original GO collection and assessed to what extent outputs matched the true constituents.

We analyzed the benchmark sets using three distinct methods. The Fisher exact test served as a baseline because it is the most suited statistical test for the task and because it underlies many existing gene-set analysis software packages [20]. We also performed nearest-neighbor search and nearest-neighbor decomposition. All three approaches were set to report at most five hits in ranked order and we quantified performance by computing the Jaccard Indexes of the top-ranked hits against the ground truth (Methods). When a benchmark set consisted of more than one constituent, we computed the sum of Jaccard Indexes against each constituent. (Methods). With this strategy, the lower bound of our score is zero and occurs when none of the the top hits match the description of the benchmark. The score is bounded from above by the number of underlying constituents. Effectively, the score counts the number of correct matches.

Evaluations of the benchmark data revealed strengths and weaknesses of the three methods under different scenarios (Figs 2B–2D and S1). For benchmark sets that consisted of genes from a single GO set with 50% coverage and 50% signal (Fig 2B), the Fisher method achieved a score of 1. This result corresponds to the best possible performance for this class of benchmarks, and search and decomposition matched it. We next performed evaluations on benchmarks sets consisting of more GO components. With four components, the Fisher test achieved a median score of two, meaning that it placed two of the components among the top-ranked outputs. This demonstrates that while the Fisher approach is reliable in matching inputs to a small number of constituents, it systematically omits parts of the signal (within the top-ranked outputs). The NN search method showed similar performance to the Fisher method. In contrast, the NN decomposition algorithm achieved twice the median score on complex benchmarks. In particular, the decomposition approach was able to place all the constituents of four-component benchmarks among the top five outputs. This demonstrates that the algorithm avoids reporting redundant outputs and deconvolutes complex signals into their constituents.

The search and decomposition approaches also performed well in other scenarios. In particular, decomposition consistently outperformed the Fisher test in benchmarks with multiple components, including in cases with low coverage (Fig 2C) and low signal-to-noise ratios (Fig 2D). Overall, these results indicate that nearest-neighbor based approaches are a reasonable approximation to the statistical method under the studied conditions.

To investigate performance of our knowledge-base and algorithms with real genesets, we turned to expression data from two human cell lines, eHAP and HAP. The genomes of these cells differ by a structural deletion of around 30 Mbp on chromosome 15 [21], which is known to drive differential expression (Fig 2E). The set of differentially expressed (DE) genes can therefore be thought of as a superposition of one component formed by genes on chromosome 15, plus potentially other components describing any other aberrations or expression noise. To study this system, we created a collection of gene sets grouping genes based on genomic windows of various widths, from 1 Mbp to 100 Mbp (Methods). We then studied how various computational approaches may describe the DE gene sets.

We performed a canonical gene-set enrichment analysis using hypeR, a modern software package [22]. The output of this method is a list of gene sets ranked by p-values, computed using hypergeometric tests (Fig 2F). The top hit was a gene set describing the region with coordinates 60M-100M on chromosome 15. This is indeed the gene set in the collection that best describes the two cell lines. In addition to this hit, many other sets also appeared with high levels of significance. Some corresponded to small regions within the 60M-100M interval. Such small gene sets are redundant given the first hit, but also harmless. However, within the top 10 results, there also appeared a gene set comprising all genes from chromosome 15. This result was significant from a statistical point of view, but may be potentially misleading from a genomics point of view.

Following the canonical analysis, we next applied nearest-neighbor search and decomposition. The search algorithm produced similar results to the statistical approach (Fig 2G). The top hit was again the 60M-100M region on chromosome 15, and other hits also matched outputs from hypeR. The ranking was not exactly the same as for the statistical test, and this is expected due to the technical search criteria. The hit list was capped to a small number by default. This setting for reporting only a small number of results contributed to better run-time performance as fewer overall comparisons were necessary to produce the output. The hit list also did not include statistical metrics such as a p-value, which should be computed in post-processing. This is a tradeoff between speed and completeness, but can be very attractive for data exploration. In contrast to search, the decomposition algorithm only produced a single result (Fig 2H). It thus summarized that after taking the abnormal 60M-100M region into account, the remaining DE genes do not strongly match to any other genomic region.

It should be mentioned that while this example displays opportunities for using DE gene sets to understand chromosomal aberrations, this approach is not a substitute for specialized copy-number analysis. In particular, this approach does not pinpoint the precise breakpoints for the structural rearrangement. Nonetheless, the results validate our algorithms using real data and demonstrate that fast algorithms can produce biologically interpretable outputs.

### User-driven learning incorporates domain knowledge to improve search outcomes

Our search and decomposition algorithms process text and are not specific to gene sets, so they can be applied to diverse data types. To demonstrate this versatility, we applied our approach to phenomics, specifically to the problem of mapping phenotypes across species. The mammalian phenotype (MP) and human phenotype (HP) ontologies provide vocabularies for abnormalities that can occur at the organism level [23,24]. They are used in knowledge-bases about animal models and human diseases [25,26], and translations between the two ontologies are essential ingredients to calculations that assess the relevance of animal models to human diseases [27,28]. Phenotype definitions can be treated as text and we set out to translate human annotations to the mammalian ontology.

**Fig 3:**
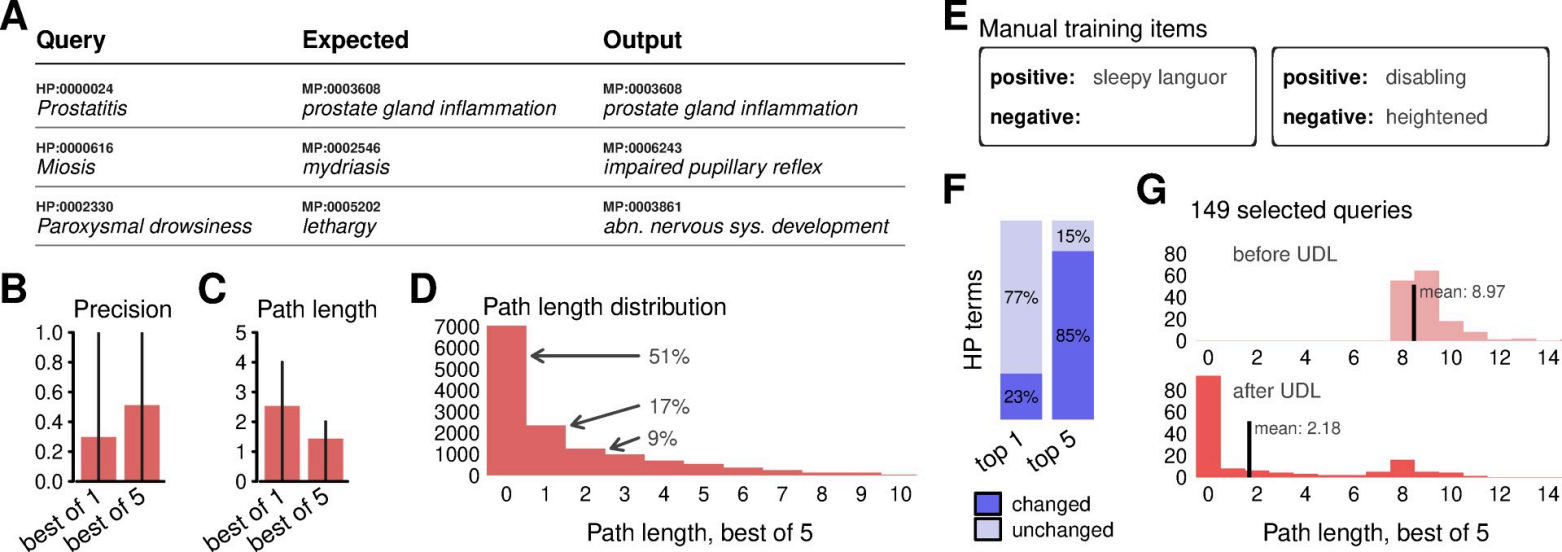
Application to ontology translation. **(A)** Examples of translations between human and mammalian phenotype ontology terms. Queries are from the HP ontology; expected outputs are MP terms suggested by an ontology-aware translation algorithm; empirical outputs are from nearest-neighbor searches. Note that while HP and MP terms are summarized by their name only, calculations rely on full term definitions. **(B)** Summary of translation precision compared with the expected mapping. Bars represent means over all HP terms, whiskers represent 25%-75% quantile ranges. **(C)** Summary of translation performance in terms of the ontology-graph distance between expected and output terms. **(D)** Distribution of path length errors. **(E)** Examples of annotations added through a graphical-user interface. **(F)** Impact of manual training items on the translation of all HP terms. **(G)** Impact of user-driven learning (UDL) on the HP terms that were targeted for training.

We prepared collections containing phenotype definitions from the MP and HP ontologies (Methods). We loaded the MP terms into a data store and performed searches using HP terms as queries. As an example, a query comprising the definition of ‘prostatitis’ from the human ontology was mapped to ‘prostate gland inflammation’ from the MP ontology (Fig 3A). This corresponds to a translation provided by an established algorithm that relies on ontology-based reasoning [29] and demonstrates that nearest-neighbor search can provide translations even when the terms are not literal analogs. However, other cases were not as concordant (Fig 3A). Averaged over all the terms in the HP ontology, the proportion of empirical results that exactly match expected outputs was 29% for the first-ranked search output and rose to 51% when using any one of the top five search results (Fig 3B). Given the relatively low apparent performance, we quantified errors in more detail using the path distance between our search results and the expected terms in the MP ontology graph. With this metric, path lengths between exact matches are zero, between child or parent terms are equal to one, between grandparents and siblings are two, and so on. The mean path length for first-ranked search results was 2.5, falling to 1.4 when considering any one of the top-five search results (Fig 3C). The distribution of path lengths had a large skew and 77% of outputs had path length at most equal to two (Fig 3D). This reveals that the majority of translations actually corresponded to similar concepts in the phenotypic space.

Stratification of HP terms revealed that items that have similar titles to MP terms achieve very high precision and low errors (S2 Fig). This suggests that HP queries might not map to expected MP terms because they encode information in different feature sets, for example, using human-centric terminology like ‘arm’ as opposed to mammalian ‘limb’, or using synonyms such as ‘elevated’ and ‘heightened’. We thus investigated data diffusion as a mechanism to alleviate such discrepancies. Data diffusion uses an ensemble of data to estimate co-occurrence of features, and then imputes missing features (Methods). After calibrating the strength of diffusion for the HP-MP translation (S3 Fig), overall precision increased to 31% and 53% as measured by the first-ranked and best-of-five results, respectively. This indicates that diffusion is constructive, but the modest improvement suggests that the corpus we used to inform the diffusion process is incomplete or insufficient to bridge the ontologies.

One of the key capabilities of our application is to accept additional data items at run-time in order to tune search results. To demonstrate this, we investigated HP terms that achieved a path-length error of at least 8 in our previous attempts. We inspected these candidates to ensure that the expected mammalian MP-terms indeed corresponded to the definitions of the human HP-terms. As an example, one of these terms was ‘paroxysmal drowsiness’, a phenotype of the nervous system, which was by default mapped to ‘abnormal nervous system development’ instead of the expected behavior phenotype ‘lethargy’, leading to a high path-length error of 9. To adjust such mappings, we manually reviewed each case and inserted new text snippets into the data store using an interactive graphical user interface. Each snippet consisted of a small number of words indicating similarity or dissimilarity (Fig 3E) and was chosen to drive diffusion in an appropriate direction (S3 Fig). After incorporating the new data items into the diffusion process, we observed substantial effects on the outputs. 23% of all HP queries were mapped to a different top hit in the MP ontology, and 85% resulted in a different set of top-five hits (Fig 3F). The impact on overall precision was small (top 5 precision increased from 53% to 55%), but the distribution of path lengths among the targeted queries displayed a pronounced shift toward lower values (Fig 3G). Among the changed mappings was ‘paroxysmal drowsiness’, which was remapped to ‘abnormal alertness’ as the top hit and ‘lethargy’ among the top five (S1 Table). This shows that users can add domain-specific knowledge and improve search results.

Understanding why individual mappings are imperfect and what adjustments are required to improve them requires manual investigation effort. In the case of translating between HP and MP ontology terms, this effort can form the beginning of an interaction with ontology curation teams to clarify or to disambiguate certain term definitions. Irrespective of changes to the underlying data sources, user-driven learning empowers users to adapt the knowledge-base to suit specific needs in real-time, without the need to retrain or rebuild a complex model. Data items added at run-time can be reused or shared amongst community members.

### Multi-modal queries combine structured and unstructured information

Beyond applications that rely on a single type of data, our software can serve as a general-purpose data integration platform. To investigate this context and demonstrate multi-modal queries, we loaded a new data store with data on gene sets, phenotypes, and human genetic diseases (ORPHANET). We then searched this knowledge-base with basic queries and then with customized configurations, including diffusion processes powered by signaling pathway annotations and protein-protein interactions. All actions were performed using a graphical user interface and thus represent queries that can be carried out interactively (Methods).

**Fig 4:**
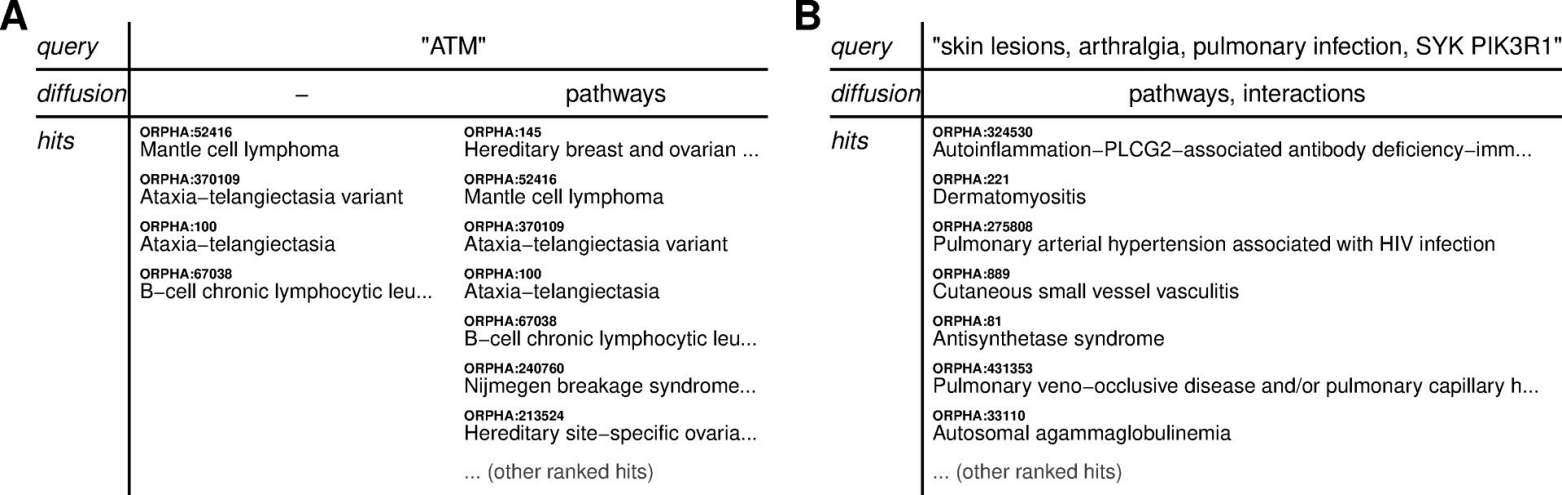
Examples of queries against a disease knowledge-base. **(A)** Results from a single-gene query with two diffusion settings. Hits are presented in ranked order. **(B)** Results from a multi-modal query with multiple phenotypes and genes.

A common use-case for disease knowledge-bases is to search for single gene symbols in order to retrieve diseases associated with defects in those genes. To illustrate this behavior, we searched for *ATM*—a gene named Ataxia Telangiectasia Mutated. This query returned four genetic diseases, including Ataxia Telangiectasia, a neurodegenerative disease after which the gene is named (Fig 4A). While this result can be obtained from canonical knowledge-bases, we then performed a related query using the same gene, but activating a diffusion process using pathway annotations. The modified query returned many more disorders (Fig 4A), including cancers that are not explicitly associated with *ATM*. This is consistent with the known roles of *ATM* in regulating the cell cycle and responding to DNA-damage. This demonstrates that diffusion can extend search results in biologically meaningful ways.

As a second example, we constructed a multi-modal query (Fig 4B). Our query included a skin, skeletal, and immunological phenotype, and two gene symbols. We set diffusion by signaling pathways and protein-protein interactions. The top hit was an immunodeficiency disease caused by *PLCG2*, phospholipase C gamma 2, a gene coding for a transmembrane signaling enzyme. This disease was among the top hits when searching only for the phenotypes. Interestingly, the match was based on a free-text description of the disease rather than a curated list with phenotype ontology codes. The disease was then ranked first after including *SYK* and *PIK3R1*, two genes that interact with *PLCG1*. Thus, the search mechanism was able to utilize pathway information to modulate the results ranking.

These case studies can be contrasted with the capabilities of existing knowledge-bases and web-search engines. Knowledge-bases excel at extracting diseases based on short queries such as gene symbols [3]. However, they do not provide control over how to broaden the scope of search to imperfect matches, which is important for explorative research. Their focus on outputting high-fidelity matches also implies that long queries can produce no hits at all; this occurs for our multi-modal example involving phenotypes and genes [3,30]. Web-search engines are popular tools because they can retrieve imperfect matches, even with very long queries. However, they do not allow users to limit search to particular datasets or to search private datasets. Similarly to knowledge-bases, they do not provide explicit mechanisms to modulate rankings, correct errors, or suggest improvements. Overall, our search approach that combines general-purpose search, diffusion, and user-driven learning thus extends the capabilities that are currently available to researchers.

## Discussion

This work investigated the utility of general-purpose tools for studying heterogeneous data. The motivation for using a simple tool for several applications, bypassing the benefits of domain-specific optimization, is that this strategy may encourage more versatile data integration and software re-use across many datasets. We focused on text-based data and the need, on the one hand, to perform calculations in a transparent manner, and, on the other hand, to adapt results given expert knowledge. We presented a practical implementation of a tool that addresses these criteria.

The software relies on well-known components—a database and a nearest-neighbor index. Two calculations demonstrate that these components can yield competitive results compared to established, domain-specific methods. We showed that nearest-neighbor search can match sets of genes to GO processes with comparable precision to accepted analysis methods, and that an iterative decomposition can outperform in certain biologically-relevant scenarios that do not satisfy the assumptions underlying the Fisher test. We also showed that the same software can map phenotypes across two ontologies and yield results that are close to translations produced by a specialized algorithm. These calculations suggest that a single framework can indeed form the backbone for nontrivial data exploration.

While our requirement to use a single framework constrains opportunities to hard-code domain-specific optimizations, it also creates an automatic mechanism to integrate distinct data collections. By treating all data as text, our search and decomposition algorithms can process not only gene sets or phenotype descriptions, but any combinations thereof. We demonstrated the possibilities for multi-modal queries in the context of matching genetic and phenotypic data to disease descriptions. The flexibility of text queries encourages interactive data exploration. This is in contrast to simple search engines that can only accept short query text, or with sophisticated knowledge-bases that require keywords, ontology identifiers, or careful query syntax.

Using a single framework for many datasets also opens opportunities to repurpose algorithms from one domain to another. Diffusion processes are used in gene-bases analyses, for example to prioritize disease-causing genes [31], and to overcome challenges with data sparsity in single-cell transcriptomics [14,32,33]. We adapted a diffusion algorithm for k-merized text and showed that this interpretable process can change rankings in nearest-neighbor searches. Importantly, we demonstrated that it is possible for expert knowledge to modulate diffusion to improve search results. To our knowledge, this is the first example of a bioinformatic tool that can learn from its users in real-time and personalize its results. The framework satisfies criteria of interpretability [34], including the ability to correct erroneous outcomes. As such, it is suited to curating biological data and enabling human-machine cooperation [35].

## Methods

### Data store with nearest-neighbors index

Our framework is implemented as a data store coupled to nearest-neighbor index (Fig 1). An instance is built by transferring one or more data collections into a mongoDB database [36] and nmslib data indices [16]. The build process consists of three stages.

A first scan splits text into words separated by spaces, and then into overlapping strings of length at most equal to an integer *k*. These strings are interchangeably called k-mers and features. The first pass through the data estimates the frequency of all k-mers across all documents. Each k-mer is assigned a weight *w* = *c*_0_−*c*_1_*log*_10_(*n*/*N*), where *n* is the number of objects containing the k-mer, *N* is the total number of objects, and *c*_0_ and *c*_1_ are user-specified constants. This approach is compatible with treating all features with equal importance (*c*_1_ = 0, suited to process unweighted gene sets) and inverse frequency (*c*_0_ = 0, suited to process natural language text that contain frequent uninformative words such as ‘the’ or ‘a’).

A second pass of the build process transfers raw data into a database in its original format as well as in a numeric encoding using sparse vectors. Words in data items that are shorter than *k*—equivalent to k-mers—are weighted by their log-frequency in the data item and by the k-mer weight. Words that are longer than *k* are additionally weighted to account for the fact that overlapping k-mers share content. Exact details are available in the software implementation. After k-merization and weighting, data items are normalized to unit norm before storing in the database. Importantly, during this stage of the build process, data can be pooled together, or arranged into distinct collections.

The final stage of the build process uses the sparse vectors to construct auxiliary data structures. One of these is an approximate nearest-neighbor index [16]. A separate data structure tracks co-occurrence of k-mers within documents. Conceptually, this structure is a square matrix *C* with elements *c*_*ij*_ that track co-occurrence of k-mers with indexes *i* and *j*. In the database, the information is stored row-wise in sparse vectors. The structure can be incremented as data items are inserted into the database. The increment is weighted by the inverse length of the data items. Thus, co-occurrence of two k-mers in a short data item is recorded with higher weight than the co-occurrence of the same two k-mers in a data item that also has many other k-mers.

The build process can be tuned by adjusting the k-mer length, total number of k-mer features, and other settings. All are described in the online software documentation.

### User-driven learning

After a knowledge-base is built, new data can be inserted to enable user-driven learning. All new data are stored in separate collections and indexes, automatically separating original- and user-level contributions. This is useful to track data provenance. It also guarantees that indexes for large data collections generated during the build process do not need to be re-generated.

Data inserted at run-time are copied into three locations. First, the data in unprocessed form are inserted into the mongoDB database. The insert operation takes constant time. Second, new data are used to increment the co-occurrence data structures. This operation requires read and update operations; the running time is proportional to the length of the new data. Third, new data are inserted into a nearest-neighbors index. In the current implementation, this requires re-generating the index. This is not problematic for small user collections, including all examples described in this work, but this step is slower-than-linear in the total number of items and can become a limiting factor if user-driven collections grow to thousands of items. However, if user-collections were restricted to drive diffusion and not for searching, this step could be eliminated altogether.

### Data diffusion

Because most user queries are expected to turn into sparse vectors, it is possible that they may not have many features in common with data items in the database, which consist of sparse vectors themselves. Data imputation can be beneficial in such situations because it can suggest non-zero weights for missing features and thus help search algorithms produce nontrivial rankings. A canonical approach to imputation would suggest values for all missing features, thus turning a sparse vector into a dense vector. Data diffusion, in contrast, is an approach that preserves sparsity by imputing only a subset of features.

Our algorithm acts on text from user queries and imputes features that have previously co-occurred with that input. Intuitively, a feature (k-mer) absent in a user’s query is assigned a high value if it co-occurred with text from the query, and is kept at zero if it has not co-occurred at all. As a result, a sparse vector representing a user’s query is kept sparse after diffusion. In practice, the implementation of data diffusion contains some subtleties. One is related to the diverse weights (importance) assigned to individual features. Our knowledge base allows common k-mers like ‘the’ to have lower importance than a rarely-used feature like ‘atrophy’. The diffusion algorithm prevents un-informative features from distorting the imputed feature by using harmonic scaling. Another detail pertains to negative weights. Whereas physical diffusion that motivates the algorithm only considers positive concentrations, our data diffusion algorithm accepts data vectors with both positive and negative values.

More technically, diffusion can be formulated as a matrix *D* with elements defined as

$$D_{ij}={\hat{c}}_{ij}\frac{2w_{i}w_{j}}{w_{i}+w_{j}}$$

Here, ${\hat{c}}_{ij}$ is a normalized entry from the co-occurrence matrix *C*. The normalization is carried out row-wise and ensures that diagonal entries in the co-occurence are set at unity. The ratio defines a harmonic mean of k-mer weights, which ensures that informative k-mers participate more in diffusion than non-informative k-mers, and that diffusion from informative k-mers does not inflate values associated with non-informative k-mers. A diffusion transformation on a query vector *q* can be formulated as

q→q+sDq

where *s* is a user-specified scalar interpreted as the strength of diffusion. This formulation automatically accommodates queries and diffusion matrices with negative entries.

In practice, the diffusion process is implemented in terms of sparse vectors instead of matrices. When the database manages multiple datasets, the diffusion transformation is extended with additional terms, i.e. with a separate user-specified strength scalar and a diffusion matrix based on co-occurrence patterns in each dataset. In order to allow interactions across datasets, the transformations are applied in two passes. When calculations are performed in sequence, the sources of diffusion are restricted to those k-mers that are present in the original query. Thus, features imputed due to diffusion based on one dataset are not used to source diffusion based on a second dataset. This restriction is sometimes termed as 1-step diffusion [14,33]. This restriction is implemented for computational efficiency and to avoid over-smoothing associated with multi-step diffusion [33].

### Search and decomposition

The primary algorithms for querying the data store are search and decomposition. Both accept inputs as strings of free text and can be augmented by data diffusion.

The search algorithm parses a query into k-mers, encodes them into a numeric vector using feature weights, and performs a lookup of nearest-neighbors. If the data store was built by separating data into separate collections, search can use any one of these collections.

The decomposition algorithm provides an alternative view of the data. It parses a query and encodes it into a numeric vector, but it then returns a small set of data items that can be used, in a linear combination, to approximately reconstruct the input. Decomposition is not a core feature of the nearest-neighbor data index and is implemented via an iterative algorithm. First, a simple search is used to find the item in the data store that best matches a query. Second, a residual vector is computed by subtracting the search result from the data query. Third, the residual vector is used to search for another item in the data store. The algorithm then iterates through this cycle, each time using a residual vector computed from a linear combination of data vectors found in previous steps. The procedure stops when the required number of outputs is obtained, or when the coefficients in the linear combination reach zero. The solution produced by the greedy algorithm is not unique and cannot be considered to be an optimal decomposition, but the running time is asymptotically comparable to that for a single search.

### Software implementation

The software is available as a python application called ‘crossmap’. The software includes a utility ‘crossprep’ to prepare data files starting from several input data formats, for example gene sets and ontology definitions.

The software includes a graphical user interface in a chat format accessible through a web browser. The interface enables users to carry out search and decomposition queries, as well as tune the diffusion process. Additional features include previewing data stored in the database, exploring how diffusion imputes new features for any input query, adding new data items, and explaining why queries map to the provided hits and not to another target.

### Example: benchmarking with gene sets

For benchmarking calculations, gene sets were downloaded from the Gene Ontology consortium. Gene sets were excluded if they were not reported under the biological process branch of the ontology, or if they contained fewer than five or more than 100 genes. One data store was loaded with gene sets using *k* = 6 as the length of k-mers and using uniform weighting (Table 1). Another data store was prepared with the same data using information-content weighting for comparison.

**Table 1:** Characteristics of crossmap instances. Build times are approximate measures obtained on a quad-core laptop with 2.7Ghz processor and 8 GB of RAM. Peak memory use are approximate measures observed during the build process; subsequent run-time memory use is typically far lower. Number of items count total data items inserted into the database. Number of features refers to the distinct k-mers. (*) For the instance with MP phenotypes, the number of items excludes any items inserted at run-time as part of user-driven learning. The number of features is exactly 200,000 as this is a default cutoff, which can be changed when needed.

Example	Build time	Peak memory use	Num. items	Num. features
GO gene sets	1 min	0.7 GB	7,227	22,147
Genes inc. GENCODE	10 min	4.2 GB	79,344	34,455
MP phenotypes*	4 min	1.3 GB	12,838	200,000
Genes and diseases	13 min	2.4 GB	90,887	469,298

Synthetic gene sets to be used for benchmarking were generated by picking genes from the GO sets according to a combination of recipes (Fig 2A). One recipe varied the number of GO sets used to source the gene set. A second recipe varied the proportion of genes transferred from the GO sets into the synthetic sets. The last recipe supplemented each synthetic set with varying amounts of randomly-selected genes. Each synthetic set was used as a query for search and decomposition. Each set was also compared against the database using the Fisher test. The top five results from each query were recorded.

Results from search, decomposition, and the Fisher test were evaluated using a score based on the Jaccard similarity. For queries where the synthetic set was drawn from one GO set, the first score was defined as the Jaccard index between the first-ranked result and the ground truth. For queries where the synthetic query was drawn from more than one GO set, the score was generalized as the sum of Jaccard indexes against the expected GO sets: the first-ranked hit was compared to one set in the ground-truth, the second-ranked hit was compared to the next component in the ground-truth, and so on for all the components in the ground truth. Overall, the score can be interpreted as the number of hits at the top of a ranked list of results. Non-integer scores are possible when the reported hits have nontrivial Jaccard overlap with the ground truth.

### Example: identification of genomic regions through gene sets

For analyses of genomic regions, gene annotations were downloaded from the GENCODE consortium [37]. Genes were partitioned into gene sets using overlapping genomic titles of sizes ranging from half a megabase to 50 megabases. The resulting gene sets acquired varying numbers of genes depending on the tile size and the density of genes along the chromosomes. Gene sets were loaded into a data store using *k* = 6 as the k-mer length and using uniform feature weighting (Table 1).

### Example: translation of phenotypes

For calculations involving ontology terms translations, ontology definitions were obtained from the OBO foundry (www.obofoundry.org). Data collections were prepared by parsing ontology obo files and extracting term titles, definitions, comments, synonyms, titles of parent terms, and the title of the top ancestor term. These components associate each term to information about its meaning and to its position in the ontology structure. Scripts to prepare such data collections from OBO ontology files are available alongside the primary crossmap software.

In order to capture the relative frequency of k-mers in natural language, another auxiliary dataset was prepared using word definitions from the wiktionary (www.wiktionary.org). Scripts to prepare data collections from the wiktionary are also available with the crossmap software.

A crossmap data store was created by loading the MP ontology and wiktionary datasets. The k-mer length was set at *k* = 6 and feature weighting, unlike in the examples with gene sets, was set to use information-content scaling (Table 1). Calculations were performed by using HP data items as queries. Search results were compared against a dataset of HP-MP term translations produced by owltools [29] and using the graph of hierarchical relations in the MP ontology.

### Example: exploration of diseases

For demonstrations of multi-modal queries, a single knowledge base was constructed holding GO gene sets, pathway gene sets from REACTOME [38], gene-interaction gene set from STRING [39], gene function annotations from GO [19], phenotypes from HP [23], disease annotations from ORPHANET (www.orpha.net), and word definitions from the wiktionary (www.wiktionary.org) in order to learn relative word frequencies. The knowledge base was built using *k* = 8 as the length of k-mers and using feature scaling based on information content (Table 1). This knowledge-base was used for interactive data exploration.

## Supporting information

S1 Fig Benchmarking search and decomposition on synthetic gene sets. **(A)** Schematic of the procedure for the generation of synthetic gene sets. Starting from a pool of genes (dots) and a set of curated gene sets (color boundaries), the procedure entails three distinct strategies. One strategy picks genes from between one and four curated gene sets (top). Another strategy modulates the proportion of genes that are transferred from the curated sets into the synthetic sets (middle). The last strategy supplements each synthetic set with genes picked from the pool at random, modulating the signal-to-noise signal (bottom). The schematic is an exact repeat of a figure panel in the main manuscript. **(B)** Summary of performance of Fisher, search, and decomposition algorithms on an ensemble of 64,000 synthetic gene sets generated according to the schematic in (A). The evaluation metric is the sum of Jaccard Index (JI): for a benchmark set made up of n components, this metric takes the n top-ranked hits from each method, evaluates the JI between the hits and the true curated sets, and reports the sum of the JI values. Effectively, the total JI counts the number of expected sets found. Box plot bounds, center line, and whiskers represent 25%-75%, 50%, and 5%-95% quantiles. Boxes contain results from all benchmark sets generated according to a mixture of strategies. Rows stratify the data according to number of components, coverage, and signal. Columns show results computed using crossmap instances using uniform feature weighting and feature weighting based on information content (IC). In the latter, genes that appear in many gene sets are down-weighted as non-informative. The Fisher method does not utilize weighting, so Fisher results are exactly the same in the two columns. **(C)** Effect of diffusion on search and decomposition performance. In these calculations, diffusion appears to lower performance slightly.
(TIF)

S2 Fig Stratification of HP-MP phenotype translation. **(A)** Examples of human phenotype (HP) terms (column ‘Query‘) and expected mammalian phenotype (MP) translations (column ‘Expected’) for which the phenotype titles are exact lexical matches. **(B)** Precision and path-length metrics for search-based phenotype translation on the subset of HP terms for which phenotype titles have exact lexical matches to MP terms. Both metrics are evaluated using the top search result and using the best choice out of the top 5 search results. Precision is not perfect (below unity) because the automated translation is based on the full phenotype description, including synonyms and comments, which are not lexically similar to MP terms. Error bars represent 5%-95% quantiles. **(C)** Distribution of path lengths for translations of HP terms that have exact lexical matches to MP terms. The distribution is dominated by exactly-correct translations with path-length of zero. **(D-F)** Analogous to (A-C), but using the subset of HP terms that do not have exact lexical matches to MP terms based on phenotype title. Panel (D) illustrates that ‘expected’ MP translations do not always capture the full meaning of the human phenotypes. Panel (F) shows that search nonetheless produces translations that match the expected results for almost half the queries. The long tail contributes to lower precision and high path-length scores for this group of HP terms.
(TIF)

S3 Fig Diffusion processes for human-to-mouse phenotype translation. **(A)** Top features imputed by a diffusion process starting from a query with text ‘activity’. Diffusion transfers weight from k-mers present in the query to other k-mers associated with enzymes. (k-mers within the query retain large weights, but are omitted from the figure to emphasize the ranking of the newly-imputed features.) **(B)** Calibration of the strength of diffusion for HP-MP translation. Queries from the HP ontology were diffused with different strengths, and then mapped to MP terms using the search algorithm. Precision was measured against mappings produced by owlsim. The shaded area of the graph is reproduced with a zoomed scale on top. **(C)** Analogous to (B), but measuring the effect of diffusion in terms of path length. **(D)** Top features imputed for the query ‘activity’ as in (A), but comparing weights obtained with plain diffusion and diffusion driven by manual annotations. The effect of manual annotations is to change the weights for the imputed features, including the introduction features associated with locomotor movement. **(E,F)** Examples of diffusion on terms that are present only in a small number of manual annotations. Some features can appear with negative values when manual annotations specify negative weights. **(G)** Example of diffusion of an HP term titled ‘paroxysmal drowsiness’, consisting of many words. **(H)** Calibration of strengths of diffusion in terms of precision, using diffusion driven by the MP ontology and manual annotations. **(I)** Analogous to (H), but measuring the impact of diffusion in terms of path length.
(TIF)

S1 Table Complete set of HP-MP translations and evaluation metrics. Columns ‘id’ and ‘name’ refer to queries from the HP ontology. Columns ‘expected’ and ‘expected_name’ denote MP terms produced by an ontology-aware translation algorithm, owlsim. Columns ‘target’ and ‘target_name’ refer to translations produced by the search algorithm. Column ‘target_N’ lists the top 5 search results. Column ‘method’ indicates the level of data diffusion: ‘plain’ for search without diffusion, ‘diffused’ for diffusion using co-occurrence within the HP ontology, and ‘best’ for the best diffusion configuration using both MP and a manual dataset. Columns ‘precision’ and ‘pathlen’ quantify the presence of a match and the extent of disagreement between expected and target results. Columns ‘precision_bestN’ and ‘pathlen_bestN’ are analogous fields, but using the best results out of the top 5 search results.
(CSV)
