# Supplementary figures and images for: Diffusion enables integration of heterogeneous data and user-driven learning in a desktop knowledge-base
Source: PLoS Comput Biol. 2021 Aug 11;17(8):e1009283. doi: 10.1371/journal.pcbi.1009283 (PMC8382188; doi:10.1371/journal.pcbi.1009283)

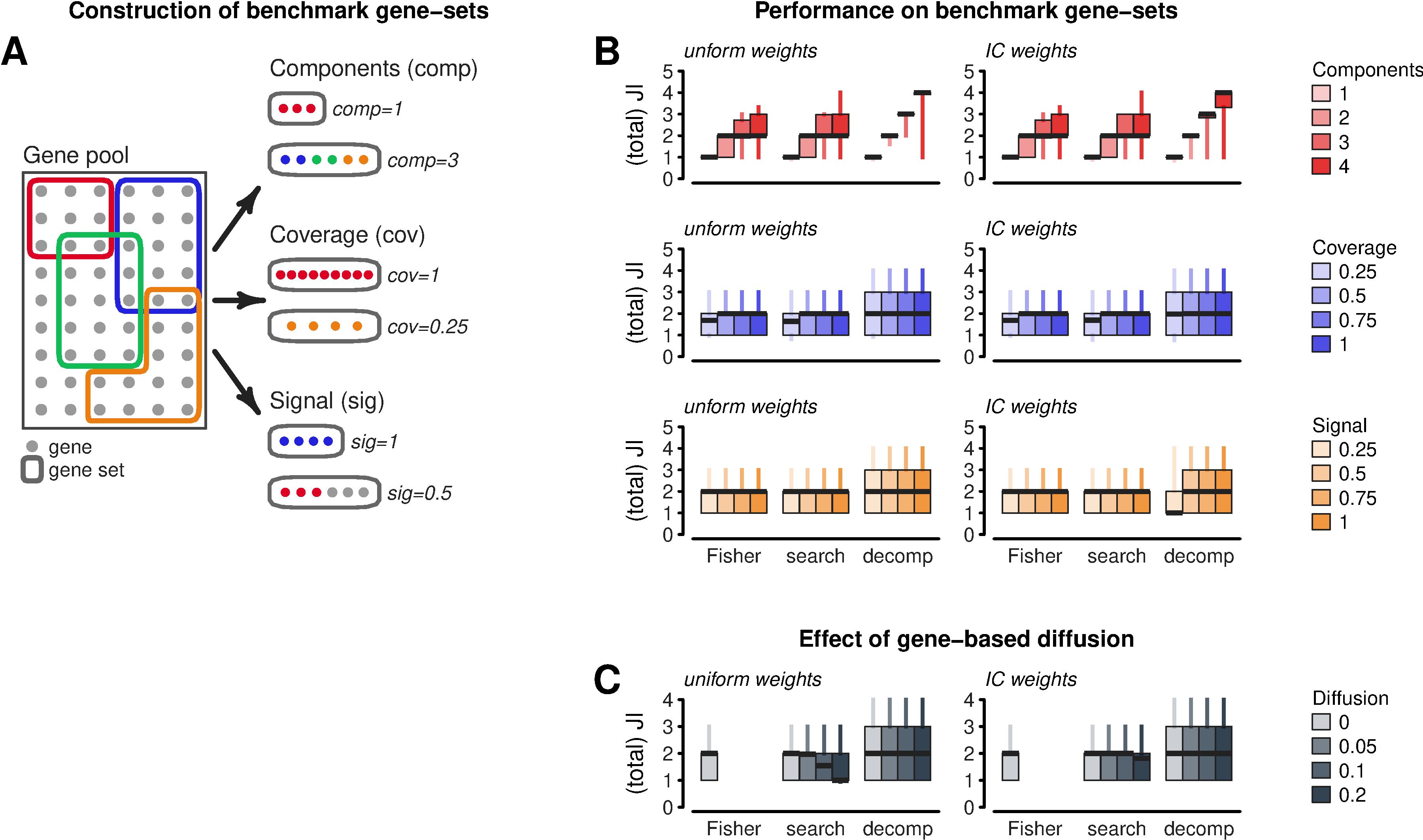

Supplement: S1 Fig — (A) Schematic of the procedure for the generation of synthetic gene sets. Starting from a pool of genes (dots) and a set of curated gene sets (color boundaries), the procedure entails three distinct strategies. One strategy picks genes from between one and four curated gene sets (top). Another strategy modulates the proportion of genes that are transferred from the curated sets into the synthetic sets (middle). The last strategy supplements each synthetic set with genes picked from the pool at random, modulating the signal-to-noise signal (bottom). The schematic is an exact repeat of a figure panel in the main manuscript. (B) Summary of performance of Fisher, search, and decomposition algorithms on an ensemble of 64,000 synthetic gene sets generated according to the schematic in (A). The evaluation metric is the sum of Jaccard Index (JI): for a benchmark set made up of n components, this metric takes the n top-ranked hits from each method, evaluates the JI between the hits and the true curated sets, and reports the sum of the JI values. Effectively, the total JI counts the number of expected sets found. Box plot bounds, center line, and whiskers represent 25%-75%, 50%, and 5%-95% quantiles. Boxes contain results from all benchmark sets generated according to a mixture of strategies. Rows stratify the data according to number of components, coverage, and signal. Columns show results computed using crossmap instances using uniform feature weighting and feature weighting based on information content (IC). In the latter, genes that appear in many gene sets are down-weighted as non-informative. The Fisher method does not utilize weighting, so Fisher results are exactly the same in the two columns. (C) Effect of diffusion on search and decomposition performance. In these calculations, diffusion appears to lower performance slightly. (TIF) [file pcbi.1009283.s001.tif]

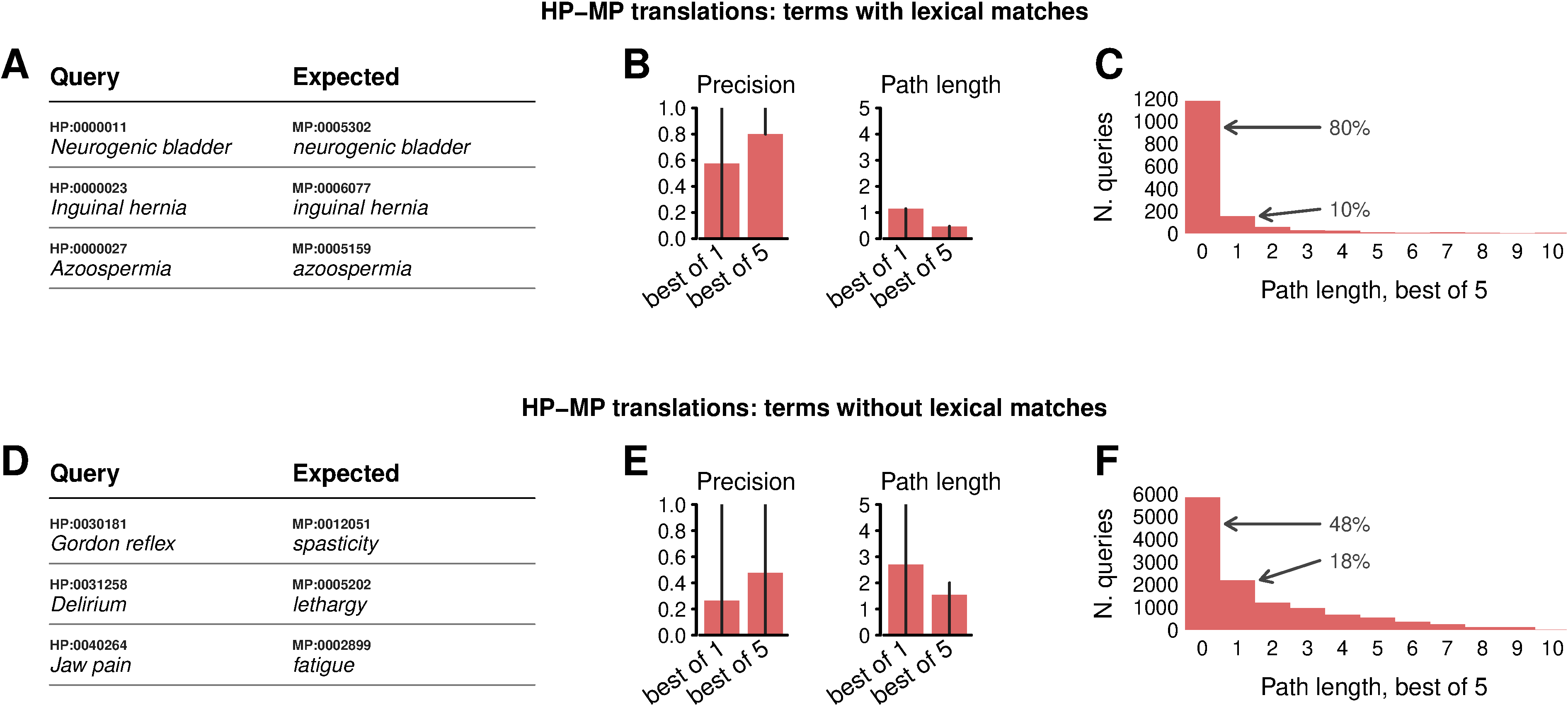

Supplement: S2 Fig — (A) Examples of human phenotype (HP) terms (column ‘Query‘) and expected mammalian phenotype (MP) translations (column ‘Expected’) for which the phenotype titles are exact lexical matches. (B) Precision and path-length metrics for search-based phenotype translation on the subset of HP terms for which phenotype titles have exact lexical matches to MP terms. Both metrics are evaluated using the top search result and using the best choice out of the top 5 search results. Precision is not perfect (below unity) because the automated translation is based on the full phenotype description, including synonyms and comments, which are not lexically similar to MP terms. Error bars represent 5%-95% quantiles. (C) Distribution of path lengths for translations of HP terms that have exact lexical matches to MP terms. The distribution is dominated by exactly-correct translations with path-length of zero. (D-F) Analogous to (A-C), but using the subset of HP terms that do not have exact lexical matches to MP terms based on phenotype title. Panel (D) illustrates that ‘expected’ MP translations do not always capture the full meaning of the human phenotypes. Panel (F) shows that search nonetheless produces translations that match the expected results for almost half the queries. The long tail contributes to lower precision and high path-length scores for this group of HP terms. (TIF) [file pcbi.1009283.s002.tif]

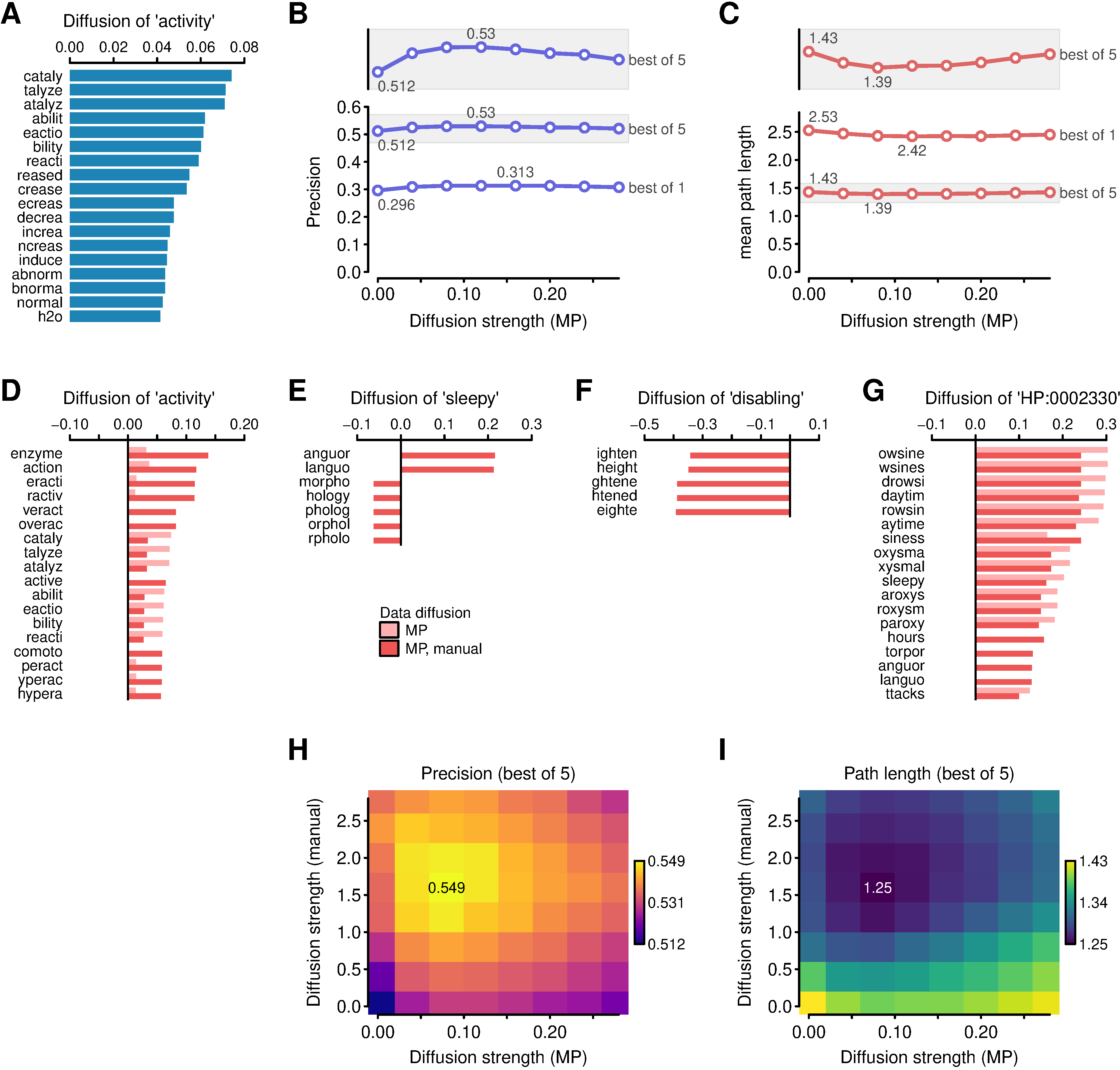

Supplement: S3 Fig — (A) Top features imputed by a diffusion process starting from a query with text ‘activity’. Diffusion transfers weight from k-mers present in the query to other k-mers associated with enzymes. (k-mers within the query retain large weights, but are omitted from the figure to emphasize the ranking of the newly-imputed features.) (B) Calibration of the strength of diffusion for HP-MP translation. Queries from the HP ontology were diffused with different strengths, and then mapped to MP terms using the search algorithm. Precision was measured against mappings produced by owlsim. The shaded area of the graph is reproduced with a zoomed scale on top. (C) Analogous to (B), but measuring the effect of diffusion in terms of path length. (D) Top features imputed for the query ‘activity’ as in (A), but comparing weights obtained with plain diffusion and diffusion driven by manual annotations. The effect of manual annotations is to change the weights for the imputed features, including the introduction features associated with locomotor movement. (E,F) Examples of diffusion on terms that are present only in a small number of manual annotations. Some features can appear with negative values when manual annotations specify negative weights. (G) Example of diffusion of an HP term titled ‘paroxysmal drowsiness’, consisting of many words. (H) Calibration of strengths of diffusion in terms of precision, using diffusion driven by the MP ontology and manual annotations. (I) Analogous to (H), but measuring the impact of diffusion in terms of path length. (TIF) [file pcbi.1009283.s003.tif]
